# Cerebrovascular reactivity is not associated with therapeutic intensity in adult traumatic brain injury: a CENTER-TBI analysis

**DOI:** 10.1007/s00701-019-03980-8

**Published:** 2019-06-25

**Authors:** Frederick A. Zeiler, Ari Ercole, Erta Beqiri, Manuel Cabeleira, Marcel Aries, Tommaso Zoerle, Marco Carbonara, Nino Stocchetti, Peter Smielewski, Marek Czosnyka, David K. Menon, the CENTER-TBI High Resolution ICU (HR ICU) Sub-Study Participants and Investigators, Audny Anke, Ronny Beer, Bo-Michael Bellander, Andras Buki, Giorgio Chevallard, Arturo Chieregato, Giuseppe Citerio, Endre Czeiter, Bart Depreitere, George Eapen, Shirin Frisvold, Raimund Helbok, Stefan Jankowski, Daniel Kondziella, Lars-Owe Koskinen, Geert Meyfroidt, Kirsten Moeller, David Nelson, Anna Piippo-Karjalainen, Andreea Radoi, Arminas Ragauskas, Rahul Raj, Jonathan Rhodes, Saulius Rocka, Rolf Rossaint, Juan Sahuquillo, Oliver Sakowitz, Ana Stevanovic, Nina Sundström, Riikka Takala, Tomas Tamosuitis, Olli Tenovuo, Peter Vajkoczy, Alessia Vargiolu, Rimantas Vilcinis, Stefan Wolf, Alexander Younsi

**Affiliations:** 1 Division of Anaesthesia, Addenbrooke’s Hospital, University of Cambridge, Cambridge, UK; 2 Department of Surgery, Rady Faculty of Health Sciences, University of Manitoba, Winnipeg, MB R3A 1R9 Canada; 3 Brain Physics Laboratory, Division of Neurosurgery, Department of Clinical Neurosciences, Addenbrooke’s Hospital, University of Cambridge, Cambridge, UK; 4 Department of Intensive Care, Maastricht UMC, Maastricht, Netherlands; 5 Neuro ICU Fondazione IRCCS Cà Granda Ospedale Maggiore Policlinico, Milan, Italy; 6 Department of Physiopathology and Transplantation, Milan University, Milan, Italy; 7 Institute of Electronic Systems, Warsaw University of Technology, Warsaw, Poland; 8 Department of Human Anatomy and Cell Science, Rady Faculty of Health Sciences, University of Manitoba, Winnipeg, Canada; 9 Department of Biomedical Engineering, Faculty of Engineering, University of Manitoba, Winnipeg, Canada

**Keywords:** Cerebrovascular reactivity, PRx, TBI, Therapeutic intensity, TIL

## Abstract

**Background:**

Impaired cerebrovascular reactivity in adult traumatic brain injury (TBI) is known to be associated with poor outcome. However, there has yet to be an analysis of the association between the comprehensively assessed intracranial hypertension therapeutic intensity level (TIL) and cerebrovascular reactivity.

**Methods:**

Using the Collaborative European Neuro Trauma Effectiveness Research in TBI (CENTER-TBI) high-resolution intensive care unit (ICU) cohort, we derived pressure reactivity index (PRx) as the moving correlation coefficient between slow-wave in ICP and mean arterial pressure, updated every minute. Mean daily PRx, and daily % time above PRx of 0 were calculated for the first 7 days of injury and ICU stay. This data was linked with the daily TIL-Intermediate scores, including total and individual treatment sub-scores. Daily mean PRx variable values were compared for each TIL treatment score via mean, standard deviation, and the Mann *U* test (Bonferroni correction for multiple comparisons). General fixed effects and mixed effects models for total TIL versus PRx were created to display the relation between TIL and cerebrovascular reactivity.

**Results:**

A total of 249 patients with 1230 ICU days of high frequency physiology matched with daily TIL, were assessed. Total TIL was unrelated to daily PRx. Most TIL sub-scores failed to display a significant relationship with the PRx variables. Mild hyperventilation (*p* < 0.0001), mild hypothermia (*p* = 0.0001), high levels of sedation for ICP control (*p* = 0.0001), and use vasopressors for CPP management (*p* < 0.0001) were found to be associated with only a modest decrease in mean daily PRx or % time with PRx above 0.

**Conclusions:**

Cerebrovascular reactivity remains relatively independent of intracranial hypertension therapeutic intensity, suggesting inadequacy of current TBI therapies in modulating impaired autoregulation. These findings support the need for investigation into the molecular mechanisms involved, or individualized physiologic targets (ICP, CPP, or Co2) in order to treat dysautoregulation actively.

**Electronic supplementary material:**

The online version of this article (10.1007/s00701-019-03980-8) contains supplementary material, which is available to authorized users.

## Introduction

Impaired cerebrovascular reactivity after traumatic brain injury (TBI) has emerged as a meaningful independent factor associated with mortality and poor functional outcome at 6 and 12 months post-injury [5, 20, 27, 28]. Current methodology for continuous measurement of cerebrovascular reactivity employed in the intensive care unit (ICU) is based on calculation of a moving correlation between slow-wave vasogenic fluctuations pulsatile cerebral blood volume (using the surrogate of intracranial pressure (ICP)), and a measure of driving pressure (such as mean arterial pressure (MAP)) [5, 26].

The pressure reactivity index (PRx), derived from ICP and MAP, is the most commonly quoted index of cerebrovascular reactivity in the TBI literature [26]. This particular index has experimental support as a potential measure of the lower limit of autoregulation [3]. Further, in adult TBI, PRx has defined critical thresholds associated with poor outcomes [20, 27].

However, despite the strong relationship between autoregulation and outcome, currently available ICU therapies do not allow us to modulate cerebrovascular reactivity as a pathway to improving outcome. A recent retrospective analysis assessed the last 25 years of experience with therapies guided by cerebral physiology monitoring in adult TBI. This analysis suggested that, despite changes in Brain Trauma Foundation (BTF) ICP and cerebral perfusion pressure (CPP) targets, little to no impact on PRx was seen [7]. This limited change in monitored PRx occurred in parallel to a stable mortality level in the same cohort over the 25-year period. This corroborates similar findings from a smaller study of 48 TBI patients, evaluating factors impacting the ability to calculate CPP optimum from PRx [24]. While these findings suggest that PRx is independent of current TBI therapies, they require robust corroboration in an analysis that accounts for the intensity of therapy. If validated, they suggest the need for further work on the molecular mechanisms or complex individual physiology involved in impaired cerebrovascular reactivity, so as to aid in the development of targeted therapies aimed at enhancing autoregulatory reserve, with the aim of increasing resilience to physiological insults and improving outcomes.

The goal of this project was to assess the association between daily treatment intensity, as measured through the therapeutic intensity level (TIL) intermediate scoring system [12, 31], and daily measures of cerebrovascular reactivity, using the Collaborative European Neuro Trauma Effectiveness Research in TBI (CENTER-TBI) study high-resolution ICU sub-study cohort [13].

## Methods

### Patient population

All patients from the multi-center CENTER-TBI high-resolution ICU cohort were included for this study. These patients were prospectively recruited between January 2015 and December 2017, from 21 centers in the European Union (EU). All patients were admitted to ICU for their TBI during the course of the study, with high-frequency digital signals recorded from their ICU monitors during the course of their ICU stay. All patients suffered predominantly from moderate to severe TBI (moderate = Glasgow Coma Score (GCS) 9 to 12, and severe = GCS of 8 or less). A minority of patients suffered from non-severe TBI, with subsequent early deterioration leading to ICU admission for care and monitoring. All patients in this cohort had invasive ICP monitoring conducted in accordance with the BTF guidelines [4].

#### Ethics

Data used in these analyses were collected as part of the CENTER-TBI study which had individual national or local regulatory approval; the UK Ethics approval is provided as an exemplar (IRAS No. 150943; REC 14/SC/1370). The CENTER-TBI study (EC grant 602150) has been conducted in accordance with all relevant laws of the EU if directly applicable or of direct effect and all relevant laws of the country where the recruiting sites were located, including but not limited to, the relevant privacy and data protection laws and regulations (the “Privacy Law”), the relevant laws and regulations on the use of human materials, and all relevant guidance relating to clinical studies from time to time in force including, but not limited to, the ICH Harmonised Tripartite Guideline for Good Clinical Practice (CPMP/ICH/135/95) (“ICH GCP”) and the World Medical Association Declaration of Helsinki entitled “Ethical Principles for Medical Research Involving Human Subjects”. Informed consent by the patients and/or the legal representative/next of kin was obtained, accordingly to the local legislations, for all patients recruited in the core dataset of CENTER-TBI and documented in the e-CRF.

### Data collection

As part of recruitment to the multi-center high-resolution ICU cohort of CENTER-TBI [13], demographics and clinical data were prospectively collected and had high-frequency digital signals from ICU monitoring recorded throughout their ICU stay, with the goal of initiating recording within 24 h of injury. All digital ICU signals were further processed (see signal acquisition/signal processing). For the purpose of this study, the following admission demographic variables were collected: age, sex, and admission Glasgow Coma Scale (GCS, total and motor). Finally, daily total TIL and all daily TIL sub-scores were also collected for each patient for the first 7 days after injury. Of note, not all patients had a full 7 days of TIL scoring given different clinical trajectories. Appendix A provides a breakdown of the TIL intermediate scoring system and its sub-score components. CENTER-TBI data version 1.0 was accessed for the purpose of this study, via Opal database software [6].

### Signal acquisition

Arterial blood pressure (ABP) was obtained through either radial or femoral arterial lines connected to pressure transducers. ICP was acquired via an intra-parenchymal strain gauge probe (Codman ICP MicroSensor; Codman & Shurtleff Inc., Raynham, MA), parenchymal fiber optic pressure sensor (Camino ICP Monitor, Integra Life Sciences, Plainsboro, NJ, USA; https://www.integralife.com/) or external ventricular drain. All signals were recorded using digital data transfer or digitized via an A/D converter (DT9801; Data Translation, Marlboro, MA), where appropriate, sampled at frequency of 100 Hz (Hz) or higher, using the ICM+ software (Cambridge Enterprise Ltd., Cambridge, UK; http://icmplus.neurosurg.cam.ac.uk) or Moberg CNS Monitor (Moberg Research Inc., Ambler, PA, USA) or a combination of both. Signal artifacts were removed using automated methods prior to further processing or analysis.

### Signal processing

Post-acquisition processing of the above signals was conducted using ICM+. CPP was determined as CPP = MAP − ICP. Ten-second moving averages (updated every 10 s to avoid data overlap) were calculated for all recorded signals: ICP, ABP (which produced MAP), and CPP.

PRx was derived using the Pearson correlation between 30 consecutive 10 s mean values for ICP and MAP, updated every minute. Data were provided in minute-by-minute comma-separated variable sheets for the entire duration of recording for each patient.

### Data processing

Post-ICM+ processing was undertaken using R (R Core Team (2016). R: A language and environment for statistical computing. R Foundation for Statistical Computing, Vienna, Austria. URL https://www.R-project.org/). Using the date and time stamp for each minute-by-minute data point, daily summaries were provided for days 1 through 7 after injury for each patient, producing daily mean ICP, daily % time with ICP above 20 mmHg, daily % time with ICP above 22 mmHg, daily mean PRx, and daily % time spent above PRx of 0. This PRx threshold was selected given previous literature confirming its relationship with global patient outcome [20], and belief that is represents the PRx value where vascular reactivity begins becoming impaired [3], representing phase-shift between slow-waves in ICP and MAP trending below 90°. These daily physiologic measures were then linked with the daily TIL measures for statistical analysis. These data are referred to as the “day-matched” data.

A second data sheet was also produced using time-shifted data. We were interested in assessing the impact of TIL score on the following-day cerebrovascular reactivity. As such, we time-shifted the TIL and physiology data in order to assess this relationship. We refer to this data as the “time-shifted” data.

### Statistics

All statistical analysis was conducted using R and XLSTAT (Addinsoft, New York, NY; https://www.xlstat.com/en/) add-on package to Microsoft Excel (Microsoft Office 15, Version 16.0.7369.1323). Normality of continuous variables was assessed via the Shapiro-Wilks test.

Box plots were used to visualize the relationship between ICP and PRx variables across increasing TIL total and sub-scores. Using total TIL score, mean ICP, and PRx variable values were compared across increasing TIL, using the Jonckheere-Terpstra test, with 1000 permutations, with alpha set at 0.05 for this test. Similarly, mean PRx variable values were compared across each TIL sub-score, with alpha set at 0.0005 after the Bonferroni correction for multiple comparisons. Given that almost all TIL sub-scores failed to demonstrate statistical significance in association with PRx variables, only the statistically significant associations are reported in detail in the manuscript. Identical results were found for mean daily PRx, and mean daily % time above PRx of 0. Similarly, identical results were found for both the day-matched and time-shifted data sheets.

In order to highlight the association between cerebrovascular reactivity with therapeutic intensity, we derived both general linear fixed effects and linear mixed effects (LME) models for total TIL versus: mean daily PRx and daily % time with PRx above 0. Patient examples were used to confirm no autocorrelation between daily physiologic measures, using autocorrelation function (ACF) and partial autocorrelation function (PACF) plots. Thus, no autocorrelative structure for the PRx variables was included in these models. Both the generalized and LME models were to be compared for superiority via the Akaike information criterion (AIC), the Bayesian information criterion (BIC), and analysis of variance (ANOVA). Alpha for ANOVA testing was set at 0.05. Given all LME and generalized fixed effects models failed to reach significance for the relationship between total TIL and cerebrovascular reactivity metrics, these models are not reported in detail within the manuscript, and only mentioned in reference to the global findings of this study.

Finally, in order to determine if there is a difference in response of PRx measures to TIL therapies during the more acute initial phase of ICU stay, we evaluated all of the above in the first 3 days of recording. Of note, none of the results were different using the first 3 days, versus the first 7 days of ICU stay. As such, we report only the first 7 days of ICU stay within this manuscript, making reference to the first 3 days analysis intermittently throughout.

## Results

### Patient demographics

**Fig. 1:**
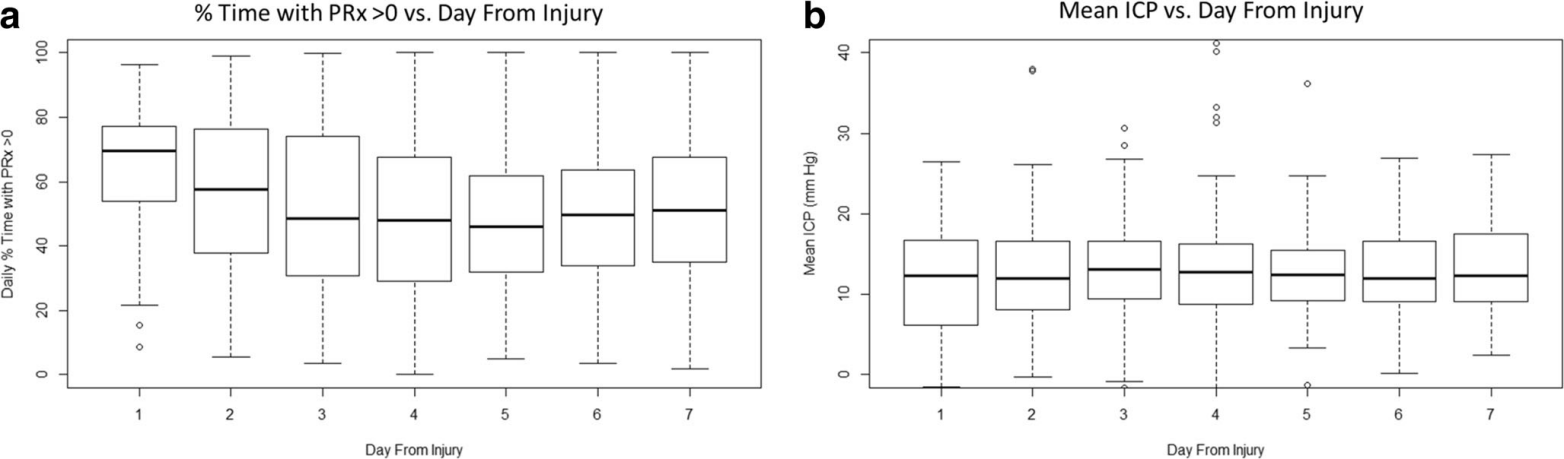
Boxplots of mean daily ICP and % time with PRx > 0 versus day. ICP, intra-cranial pressure; MAP, mean arterial pressure; mmHg, millimeter of mercury; PRx, pressure reactivity index (moving correlation between ICP and MAP). Day from injury (i.e., day-matched data), day 1 = day of injury. Plot of daily % time with PRx > 0 versus day from injury to day-matched data **a**; Plot of mean daily ICP versus day from injury to day-matched data **b**

There were 249 patients from the CENTER-TBI high-resolution ICU cohort, for whom high-frequency physiologic signals and daily TIL scores were available, and were included in this study. An account of general patient characteristics can be found in other publications on this high-resolution data cohort [28, 29]. A total of 1230 daily observations of TIL and daily PRx variables were used for this analysis. The mean age was 46.8 ± 18.9 years, with 198 being male. Median admission GCS was 6 (IQR, 3 to 10), median admission GCS motor score was 4 (IQR, 1 to 5), and median duration of physiologic monitoring was 140.6 h (IQR, 94.4 to 213.6). All continuous variables were found to be non-parametrically distributed. Mean daily ICP and PRx variable values across the patient population remained relatively constant, with mean daily ICP well below BTF defined thresholds and mean daily % time with PRx above 0 remaining consistently above 40 to 50%. There was only a significant drop in % time with PRx above zero going from day 1 (i.e., first 24 h after injury) to day 2 post-injury (*p* = 0.03; Mann *U*), and going from day 2 to day 3 (*p* = 0.004; Mann *U*), with no difference between days 3 and 7. A similar relationship was seen for % time with ICP above 20 mmHg, % time with ICP above 22 mmHg. Figure 1 displays boxplots for the mean daily ICP and mean daily % time with PRx above 0.

### Daily total TIL and cerebrovascular reactivity

**Fig. 2:**
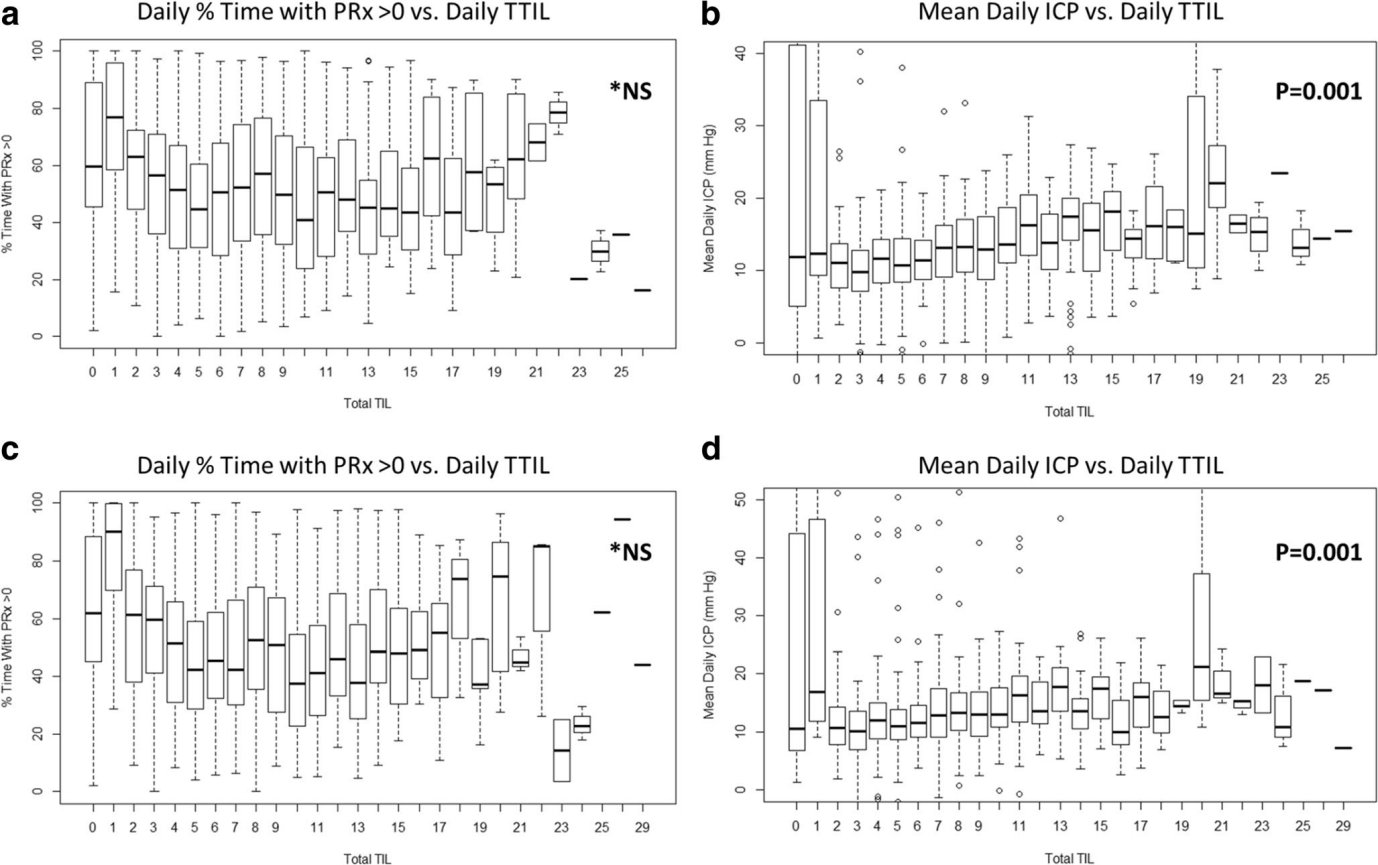
Boxplots of mean daily ICP and mean daily % time with PRx > 0 versus daily total TIL. ICP, intra-cranial pressure; MAP, mean arterial pressure; mmHg, millimeter of mercury; NS, non-significant; TIL, therapeutic intensity level; TTIL, total daily TIL. *p values reported are for the Jonckheere-Terpstra test for increasing mean values. Relationship between % time and PRx > 0 was non-significant for both increasing and decreasing mean values. Plot of mean daily % time with PRx above 0 versus daily TTIL **a**; Plot of mean daily ICP versus daily TTIL **b**; Plot of mean daily % time with PRx above 0 versus daily TTIL for time-shifted data **c**; Plot of mean daily ICP versus daily TTIL for time-shifted data **d**

Comparing total daily TIL to the daily measures of ICP and PRx, the same relationships were seen for the day-matched and time-shifted data, using both the first 7 days and first 3 days of data. With increasing total daily TIL, there was an increase in daily mean ICP, % time above ICP of 20 mmHg and % time above ICP of 22 mmHg (*p* = 0.001 on Jonckheere Terpstra testing for increasing values in all); in keeping with escalating daily therapy for increasing daily ICP measures. In contrast, there was no relationship between increasing daily total TIL and daily measures of PRx (mean values or % time above threshold; *p* > 0.05 on Jonckheere-Terpstra test for increasing and decreasing values for all), in both the day-matched and time-shifted data sheets. This suggests potentially limited treatment effect of daily TIL measured therapies on daily cerebrovascular reactivity metrics. Figure 2 displays the boxplots of total daily TIL and both daily % time with PRx above 0 and mean daily ICP, for both the day-matched and time-shifted data.

Generalized and LME models were created to assess the relationship between total TIL and mean daily PRx and daily % time with PRx above 0. All models failed to display statistically significant relationships with total TIL, even with the introduction of random effects to both the intercept and gradient by patient (*p* < 0.05 for all). As such these models will not be reported further here, but provide confirmatory evidence for the lack of association between TIL and cerebrovascular reactivity metrics.

### Daily TIL sub-scores and cerebrovascular reactivity

Comparing daily TIL sub-scores to daily measures of PRx, identical relationships were seen for all PRx variables (i.e. ,mean PRx, % time with PRx > 0), in both the day-matched and time-shifted datasets, using both the first 7 days and first 3 days of data. Only 4 daily TIL sub-score measures displayed a statistically significant decrease in daily PRx measures using day-matched and time-shifted data on Mann *U* testing: mild hyperventilation (PaCO_2_ 35 to 40 mmHg), mild hypothermia (core temp > 35 °C), high sedation levels targeting ICP, and vasopressor therapy targeting CPP goals. Table 1 provides the Mann *U* testing for % time with PRx above 0 for the 4 daily TIL sub-scores for both the day-matched and time-shifted data. Appendix B provides the boxplots for both the time-matched and time-shifted data. Of note, the overall absolute reduction in daily % time with PRx above 0 seen with these 4 interventions was less than 10%, indicating a modest potential treatment.

**Table 1:** Mean daily % time with PRx > 0, the Mann *U* testing for significant TIL sub-scores

TIL sub-score	Day-matched data		Mann *U p* value	Time-shifted data		Mann *U p* value
	Mean (± SD) daily % time with PRx > 0			Mean (± SD) daily % time with PRx > 0		
	No intervention	Intervention		No intervention	Intervention	
Fluid (vasopressors)	57.7 (23.0)	49.5 (24.0)	**2.1 × 10** ^**−8**^	57.0 (23.2)	47.8 (23.4)	**1.7 × 20** ^**−8**^
Hyperventilation (mild)	55.7 (24.6)	48.0 (22.6)	**2.5 × 10** ^**−8**^	54.5 (24.6)	46.3 (22.1)	**5.8 × 10** ^**−8**^
Hypothermia (mild)	52.8 (24.0)	44.5 (22.4)	**0.0001**	51.1 (23.9)	43.5 (21.0)	0.001
Sedation (High)	54.2 (23.4)	49.0 (24.4)	**0.0001**	53.4 (23.5)	46.7 (23.5)	**1.1 × 10** ^**−6**^

*CPP*, cerebral perfusion pressure; *ICP*, intra-cranial pressure; *MAP*, mean arterial pressure; *PRx*, pressure reactivity index (correlation between slow-waves in ICP and MAP); *SD*, standard deviation; *TIL*, therapeutic intensity level. This table reports *p* values from the Mann *U* testing, comparing mean daily % time above PRx of 0 for specific TIL sub-scores. This table reports both the day-matched data sheet and the time-shifted data sheet, evaluating the difference in mean values between those receiving a specific intervention vs. those who did not. *Note: *p* values are bolded for statistical significance after the Bonferroni correction. TIL fluid (vasopressor) refers to the need for vasopressor therapy to maintain CPP goals. TIL hyperventilation (mild) refers to mild hypocapnia for ICP control (PaCO_2_ = 35 to 40 mmHg). TIL hypothermia (mild) refers to cooling to no lower than 35 °C. TIL sedation (high) refers to high sedation levels aimed at ICP control, but not burst suppression

## Discussion

The analysis of the relationship between TIL and measures of cerebrovascular reactivity provided some interesting results which deserve highlighting. First, evaluating the % time spent with PRx above zero across the first 7 days of ICU stay, it can be seen that a large portion of any given day is spent with impaired cerebrovascular reactivity. There is statistically significant decrease in this value going from the first 24 h post injury to day 2 and day 3 in our cohort. However, from day 3 onwards, there is little change in the amount of time spent with PRx above zero, with no difference between days. As discussed below, this initial drop in time above PRx of zero is not accounted for in total TIL or specific TIL-based measures, and likely represents the initial spike in impaired cerebrovascular reactivity after the primary insult, which then improves spontaneously over the next 48 h of ICU care. With that said, the TIL metrics provided are course measures of therapeutic intensity with documentation daily in our data set. It is unknown if more frequent intervention measures, perhaps accurately marked in the high-frequency physiology data, would provide stronger associations with changes in PRx metrics over time. Recent literature supports variance in mean PRx response after TBI, with those displaying positive global outcomes having a more rapid decline in PRx during the initial ICU days of care [22]. Thus, future analysis of the impact of therapeutic measures on cerebrovascular reactivity will not only benefit from high-resolution treatment data, but also various sub-group analyses based on age, sex, co-morbidities, injury pattern/burden, and patient global outcome. Such analyses will require large multi-center data sets to accomplish these goals.

Second, therapeutic intensity as measured through the total TIL score has little relationship with cerebrovascular reactivity, either in an analysis of contemporaneous data, or when lag effects are explored in time-shifted TIL data. This was also confirmed using the first 7 days and first 3 days of data, displaying identical results, indicating no difference between the entire ICU stay versus the initial acute 72 h. These results were exemplified by the lack of statistically significant generalized and LME modeling between TTIL and all cerebrovascular reactivity metrics. This is an important finding, which suggests that overall, current ICU-based treatment strategies for TBI essentially have limited impact on cerebrovascular reactivity. This corroborates the findings of Donnelly et al. in a large single center retrospective cohort of over 1000 TBI patients, where despite changes in BTF-based ICP and CPP targets, little change in PRx or outcome was seen over 25 years [7].

Third, daily TIL sub-scores for most individual interventions failed to show any significant relationship with the PRx metrics we explored. Only four daily TIL sub-score parameters were found to be associated with lower mean daily PRx and daily % time with PRx above 0. These included mild hyperventilation, mild hypothermia, high levels of sedation for ICP therapy, and vasopressor usage to target CPP. These results were similar for both the day-matched and time-shifted data sheets. Of note, the absolute reduction in % time with PRx above 0 was only modest for these 4 treatments, with less than 10% reduction. This highlights that the majority of currently applied therapies for TBI have limited impact on cerebrovascular reactivity. With that said, it must be acknowledged that given the strong link between impaired cerebrovascular reactivity and global patient outcome documented in various patient series [5, 7, 20, 28, 29], even small changes in vascular reactivity metrics related to current TBI therapies may carry important implications for mortality and morbidity. It is still unclear how much of an improvement in PRx, or other cerebrovascular reactivity metrics, is required in order to leave a demonstrable impact of patient global outcome. Thus, despite the majority of TIL-measured therapies failing to demonstrate a significant change in PRx in this study, those interventions which had an impact may carry an important role moving forward in the initial modulation of cerebrovascular reactivity. Further to this, patient sub-group analysis based on injury pattern, injury burden, and age may play a role in individual patient response to current TBI therapies. As such, one should not walk away from this current work thinking “all is lost” regarding the impact of current TBI treatments on cerebrovascular reactivity. Much further work in this area is required.

Fourth, mild hyperventilation appeared to be associated with reduced mean daily PRx and daily % time with PRx above 0. This is in keeping with the literature body supporting improved vascular reactivity with mild hyperventilation [15, 16, 18]. However, more intensive daily hyperventilation, as assessed via other daily TIL sub-score measures (i.e., PaCO_2_ < 35 mmHg), failed to display any relationship with cerebrovascular reactivity [16]. This requires further investigation using treatment data with higher temporal resolution. However, the lack of association with cerebrovascular reactivity in the setting of more intensive hyperventilation may be secondary to exhaustion of the cerebrovascular reserve with such intensive reduction in CO_2_ levels [16].

Fifth, mild hypothermia (i.e., core temp 35–36.5 °C) was associated with a modest reduction in mean daily PRx and daily percentage of time above PRx of 0, with more intensive hypothermia therapy (i.e., core temp below 35 °C) not being associated with vascular reactivity in this cohort. Stable/improved cerebral autoregulatory capacity is known to occur with hypothermia therapy [2, 10, 11]. However, experimental models [8, 9, 23], indicate that moderate to severe degrees of hypothermia can lead to loss of cerebrovascular reactivity. This may account for the lack of association between PRx variables and more intensive measures of hypothermia measures through other TIL sub-score metrics. This intimate relation between core and brain temperature requires further investigation using continuously measured cerebrovascular reactivity.

Sixth, high levels of sedation for ICP control were associated with a modest decrease in daily mean PRx and daily % time above PRx of 0. This likely represents a treatment effect on ICP, and subsequently CPP, with CPP values being obtained closer to the optimal CPP value [1, 17, 21]. However, sedation aimed at burst suppression, as assessed via a separate TIL sub-score value, was not associated with a change in PRx metrics. This may reflect injury/disease severity, or reflect the impairment in cerebrovascular reactivity seen with burst suppression in other patient cohorts using various anesthetic agents [19, 25]. Further analysis of this relationship between sedative levels and cerebrovascular reactivity is required, using higher resolution data.

Seventh, the use of vasopressors to target CPP was also associated with a modest decrease in mean daily PRx and daily percentage of time with PRx above 0. This likely reflects improved CPP targeting with vasopressors and avoiding CPP values below optimal CPP metrics. However, the impact of vasoactive compounds on cerebrovascular reactivity after TBI is still unclear, with many leading to vasoconstrictive states in experimental models [14], and requires future investigation using high temporal resolution vasopressor dosing data linked with high-frequency physiology in time-series.

Finally, the overall daily time spent with PRx above 0 was 40% or higher for all days, during the first 7 days after injury. This highlights the unchanged burden of impaired cerebrovascular reactivity after TBI, despite ongoing active ICP and CPP treatment. It further raises the questions of the role for individualized physiologic targets in TBI care, such as those posed by individual CPP optimum therapy [1, 17, 21], which is currently under prospective evaluation. Overall, this motivates further investigation into the molecular mechanisms involved in impaired cerebrovascular reactivity after TBI. Such analysis will require the integration of protein biomarkers with genetic profiling [30], in order to determine potential mechanisms driving impaired cerebrovascular reactivity. It is through such techniques that therapies directed at prevention and treatment may be developed, potentially leading to improved morbidity and mortality in TBI.

### Limitations

The overall patient numbers with outcome and basic demographics were low at 249. This high-resolution cohort was a small specialty sub-cohort within the larger CENTER-TBI data collection scheme. This particular cohort of patients is unique in providing both high-frequency physiologic data and daily TIL scores during the acute phase of ICU stay, starting from the day of injury. Similar data will be needed to replicate our results, and more detailed assessments of the impact of interventions on autoregulatory efficiency will require therapy data with higher temporal resolution of treatment characteristics, such as TIL/TIL sub-scores, vasopressor dosing, and sedation dosing.

Furthermore, the impact of mild hyperventilation, mild hypothermia, high sedation levels, and vasopressor usage is still unclear. The impact on PRx metrics was modest at best in this analysis. Future studies require higher frequency data collection for TIL, core temperature, brain temperature, sedation dosing, and vasopressor dosing, in order to determine if any temporal response is seen between these treatments and cerebrovascular reactivity.

It must be acknowledged that TIL scores, and sub-scores, are relatively gross measures of therapeutic intensity. As such, despite failing to demonstrate a strong statistical significance with daily vascular reactivity metrics, therapeutic intensity measures captured at a higher temporal frequency, with more detailed annotations linked with the real-time physiology, may demonstrate an association with vascular reactivity measures. As such, the results of this study should be considered preliminary. Further studies are planned to evaluate the association between vascular reactivity, ICP, CPP, and other cerebral physiologic measures, using time-series physiology data with detailed treatment annotations.

Finally, in such a small data set, with various TIL-based treatment metrics measured, sometimes with very few patients have specific interventions, it raises the question of potential false-positive or false-negative results. We attempted to account for such false statistical positives through alpha correction using the Bonferonni methodology, only report results as significant with a *p* value of less than 0.0005. As such, the positive results reported, we feel are likely true positives. However, the significant reported TIL sub-scores were amongst the main drivers of overall TIL in our cohort (as seen in Appendix C). Thus, the overall significant association with PRx metrics may just be a reflection of these aspects being reported more frequently. In parallel, with such a small data set and large number of treatment variables, with a strict corrected alpha of 0.0005, there exists the potential that some of the negative results may be false-negatives. As such, we emphasize the preliminary nature of our results here, and highlight the need for larger future studies with higher frequency treatment data. The results within this manuscript should be considered exploratory only at this time.

Given all the highlighted limitations, the results of this study should be considered preliminary, despite corroborating the previous retrospective work in the literature [7].

## Conclusions

Cerebrovascular reactivity remains relatively independent of therapeutic intensity, suggesting inadequacy of current TBI therapies in modulating impaired autoregulation. These findings provide support for prior preliminary retrospective findings, and support the need for investigation into the molecular mechanisms involved in order to develop therapies aimed at prevention and treatment.

## Electronic supplementary material

ESM 1 (DOCX 15 kb).

ESM 2 (DOCX 270 kb).

ESM 3 (DOCX 31130 kb).

## References

[1] Aries MJH, Czosnyka M, Budohoski KP (2012). Continuous determination of optimal cerebral perfusion pressure in traumatic brain injury. Crit Care Med.

[2] Bisschops LLA, Hoedemaekers CWE, Simons KS, van der Hoeven JG (2010). Preserved metabolic coupling and cerebrovascular reactivity during mild hypothermia after cardiac arrest. Crit Care Med.

[3] Brady KM, Lee JK, Kibler KK, Easley RB, Koehler RC, Shaffner DH (2008). Continuous measurement of autoregulation by spontaneous fluctuations in cerebral perfusion pressure: comparison of 3 methods. Stroke.

[4] Carney N, Totten AM, O’Reilly C (2017). Guidelines for the management of severe traumatic brain injury, Fourth Edition. Neurosurgery.

[5] Czosnyka M, Smielewski P, Kirkpatrick P, Laing RJ, Menon D, Pickard JD (1997). Continuous assessment of the cerebral vasomotor reactivity in head injury. Neurosurgery.

[6] Doiron D, Marcon Y, Fortier I, Burton P, Ferretti V (2017). Software application profile: Opal and Mica: open-source software solutions for epidemiological data management, harmonization and dissemination. Int J Epidemiol.

[7] Donnelly J, Czosnyka M, Adams H et al (2018) Twenty-five years of intracranial pressure monitoring after severe traumatic brain injury: a retrospective, single-center analysis. Neurosurgery. 10.1093/neuros/nyy468 10.1093/neuros/nyy468 30476233

[8] Fujita M, Wei EP, Povlishock JT (2012) Effects of hypothermia on cerebral autoregulatory vascular responses in two rodent models of traumatic brain injury. J Neurotrauma. 2019 May 15;36(10):1505-1517. 10.1089/neu.2018.6182 10.1089/neu.2011.2278 PMC3335136 22364620

[9] Irikura K, Miyasaka Y, Nagai S, Yuzawa I, Morii S, Fujii K (1998). Moderate hypothermia reduces hypotensive, but not hypercapnic vasodilation of pial arterioles in rats. J Cereb Blood Flow Metab.

[10] Koizumi H, Suehiro E, Fujiyama Y, Yoneda H, Ishihara H, Nomura S, Fujii M, Suzuki M (2016). Effects of brain temperature on cerebrovascular autoregulation during the acute stage of severe traumatic brain injury. Acta Neurochir Suppl.

[11] Lavinio A, Timofeev I, Nortje J (2007). Cerebrovascular reactivity during hypothermia and rewarming. Br J Anaesth.

[12] Maas AIR, Harrison-Felix CL, Menon D (2011). Standardizing data collection in traumatic brain injury. J Neurotrauma.

[13] Maas AIR, Menon DK, Steyerberg EW, Citerio G, Lecky F, Manley GT, Hill S, Legrand V, Sorgner A, CENTER-TBI Participants and Investigators (2015). Collaborative European NeuroTrauma Effectiveness Research in Traumatic Brain Injury (CENTER-TBI): a prospective longitudinal observational study. Neurosurgery.

[14] McCulloch J, Edvinsson L (1984). Cerebrovascular smooth muscle reactivity: a critical appraisal of in vitro and in situ techniques. J Cereb Blood Flow Metab.

[15] McCulloch TJ, Turner MJ (2009). The effects of hypocapnia and the cerebral autoregulatory response on cerebrovascular resistance and apparent zero flow pressure during isoflurane anesthesia. Anesth Analg.

[16] Meng L, Gelb AW (2015). Regulation of cerebral autoregulation by carbon dioxide. Anesthesiology.

[17] Needham E, McFadyen C, Newcombe V, Synnot AJ, Czosnyka M, Menon D (2017). Cerebral perfusion pressure targets individualized to pressure-reactivity index in moderate to severe traumatic brain injury: a systematic review. J Neurotrauma.

[18] Newell DW, Weber JP, Watson R, Aaslid R, Winn HR (1996). Effect of transient moderate hyperventilation on dynamic cerebral autoregulation after severe head injury. Neurosurgery.

[19] Reinsfelt B, Westerlind A, Houltz E, Ederberg S, Elam M, Ricksten S-E (2003). The effects of isoflurane-induced electroencephalographic burst suppression on cerebral blood flow velocity and cerebral oxygen extraction during cardiopulmonary bypass. Anesth Analg.

[20] Sorrentino E, Diedler J, Kasprowicz M (2012). Critical thresholds for cerebrovascular reactivity after traumatic brain injury. Neurocrit Care.

[21] Steiner LA, Czosnyka M, Piechnik SK, Smielewski P, Chatfield D, Menon DK, Pickard JD (2002). Continuous monitoring of cerebrovascular pressure reactivity allows determination of optimal cerebral perfusion pressure in patients with traumatic brain injury. Crit Care Med.

[22] Svedung Wettervik T, Howells T, Enblad P, Lewén A (2019) Temporal neurophysiological dynamics in traumatic brain injury: role of pressure reactivity and optimal cerebral perfusion pressure for predicting outcome. J Neurotrauma. 10.1089/neu.2018.6157 10.1089/neu.2018.6157 30595128

[23] Verhaegen MJ, Todd MM, Hindman BJ, Warner DS (1993). Cerebral autoregulation during moderate hypothermia in rats. Stroke.

[24] Weersink CSA, Aries MJH, Dias C (2015). Clinical and physiological events that contribute to the success rate of finding “optimal” cerebral perfusion pressure in severe brain trauma patients. Crit Care Med.

[25] Woodcock TE, Murkin JM, Farrar JK, Tweed WA, Guiraudon GM, McKenzie FN (1987). Pharmacologic EEG suppression during cardiopulmonary bypass: cerebral hemodynamic and metabolic effects of thiopental or isoflurane during hypothermia and normothermia. Anesthesiology.

[26] Zeiler FA, Donnelly J, Calviello L, Smielewski P, Menon DK, Czosnyka M (2017). Pressure autoregulation measurement techniques in adult traumatic brain injury, part II: a scoping review of continuous methods. J Neurotrauma.

[27] Zeiler FA, Donnelly J, Smieleweski P, Menon D, Hutchinson PJ, Czosnyka M (2018). Critical thresholds of ICP derived continuous cerebrovascular reactivity indices for outcome prediction in non-craniectomized TBI patients: PRx, PAx and RAC. J Neurotrauma.

[28] Zeiler FA, Ercole A, Cabeleira M, Carbonara M, Stocchetti N, Menon DK, Smielewski P, Czosnyka M (2018) Comparison of performance of different optimal cerebral perfusion pressure parameters for outcome prediction in adult TBI: a CENTER-TBI study. J Neurotrauma 10.1089/neu.2018.6182 30384809

[29] Zeiler FA, Ercole A, Cabeleira M, Zoerle T, Stocchetti NN, Menon DK, Smielewski P, Czosnyka M, CENTER-TBI High Resolution Sub-Study Participants and Investigators (2019) Univariate comparison of performance of different cerebrovascular reactivity indices for outcome association in adult TBI: a CENTER-TBI study. Acta Neurochir. 10.1007/s00701-019-03844-1 10.1007/s00701-019-03844-1 PMC6525666 30877472

[30] Zeiler FA, Thelin EP, Donnelly J, Stevens AR, Smielewski P, Czosnyka M, Hutchinson PJ, Menon DK (2019). Genetic drivers of cerebral blood flow dysfunction in TBI: a speculative synthesis. Nat Rev Neurol.

[31] Zuercher P, Groen JL, Aries MJH, Steyerberg EW, Maas AIR, Ercole A, Menon DK (2016). Reliability and validity of the therapy intensity level scale: analysis of clinimetric properties of a novel approach to assess management of intracranial pressure in traumatic brain injury. J Neurotrauma.

