# Supplementary material for: Cerebrovascular reactivity is not associated with therapeutic intensity in adult traumatic brain injury: a CENTER-TBI analysis
Source: Acta Neurochir (Wien). 2019 Jun 25;161(9):1955–64. doi: 10.1007/s00701-019-03980-8 (PMC6704258; doi:10.1007/s00701-019-03980-8)
Supplement: Supplementary file 1 — (DOCX 15 kb). [file 701_2019_3980_MOESM1_ESM.docx]

Appendix A: TIL Intermediate Scoring System Summary

| **TIL Sub-Category** | **Intervention** | **If Yes** | **Maximum Score** |
| --- | --- | --- | --- |
| ***Positioning*** | Head elevation for ICP control | 1 | 1 |
|  | Nursed flat (180^o^) for CPP management | 1 |  |
| ***Sedation Level*** | Sedation (low-dose as required for mechanical ventilation) | 1 | 8 |
|  | Higher-dose sedation for ICP control (not aiming for burst suppression) | 2 |  |
|  | Metabolic suppression for ICP control with high-dose barbiturates or propofol | 5 |  |
| ***NMBA*** | Neuromuscular blockade (paralysis) | 3 |  |
| ***CSF Drainage*** | CSF drainage < 120 mL/d (<5 mL/h) | 2 | 3 |
|  | CSF drainage ≤ 120 mL/d (≤5 mL/h) | 3 |  |
| ***Fluid/Vasopressor Therapy*** | Fluid loading for maintenance of cerebral perfusion | 1 | 2 |
|  | Vasopressor therapy required for management of cerebral perfusion | 1 |  |
| ***Hyperventilation*** | Mild hypocapnia for ICP control (PaCO_2_ 4.6–5.3 kPa [35–40 mm Hg]) | 1 | 4 |
|  | Moderate hypocapnia for ICP control (PaCO_2_ ≤ 4 kPa [30 mm Hg]) | 2 |  |
|  | Intensive hypocapnia for ICP control (PaCO_2_ < 4 kPa [30 mm Hg]) | 4 |  |
| ***Hyperosmolar Therapy*** | Hyperosmolar therapy with mannitol up to 2 gm/kg/24 h | 2 | 6 |
|  | Hyperosmolar therapy with hypertonic saline up to 0.3 gm/kg/24 h | 2 |  |
|  | Hyperosmolar therapy with mannitol > 2 gm/kg/24 h | 3 |  |
|  | Hyperosmolar therapy with hypertonic saline > 0.3 gm/kg/24 h | 3 |  |
| ***Temperature Management*** | Treatment of fever (>38C) or spontaneous temperature of 34.5C | 1 | 5 |
|  | Mild hypothermia for ICP control with a lower limit of 35C | 2 |  |
|  | Hypothermia below 35C | 5 |  |
| ***Surgery for ICP Control*** | Intracranial operation for progressive mass lesion, not scheduled on admission | 4 | 9 |
|  | Decompressive craniectomy | 5 |  |
|  | ***Total maximal score:*** |  | 38 |

C = Celsius, CPP = cerebral perfusion pressure, CSF = cerebrospinal fluid, d = day, gm = gram, h = hour, ICP = intra-cranial pressure, kg = kilogram, mL = milliliter, mmHg = millimeter of Mercury, NMBA = neuromuscular blockage agent, TIL = Therapeutic Intensity Level.
