# Supplementary material for: Cerebrovascular reactivity is not associated with therapeutic intensity in adult traumatic brain injury: a CENTER-TBI analysis
Source: Acta Neurochir (Wien). 2019 Jun 25;161(9):1955–64. doi: 10.1007/s00701-019-03980-8 (PMC6704258; doi:10.1007/s00701-019-03980-8)
Supplement: Supplementary file 2 — (DOCX 270 kb). [file 701_2019_3980_MOESM2_ESM.docx]

**Appendix B: Box Plots – Statistically Significant TIL Sub-Scores - Time Shifted Data Sheet**

Appendix B: Box-Plots of Daily % Time with PRx >0 Versus Daily TIL Sub-Scores of Significance – Day-Matched Data

**
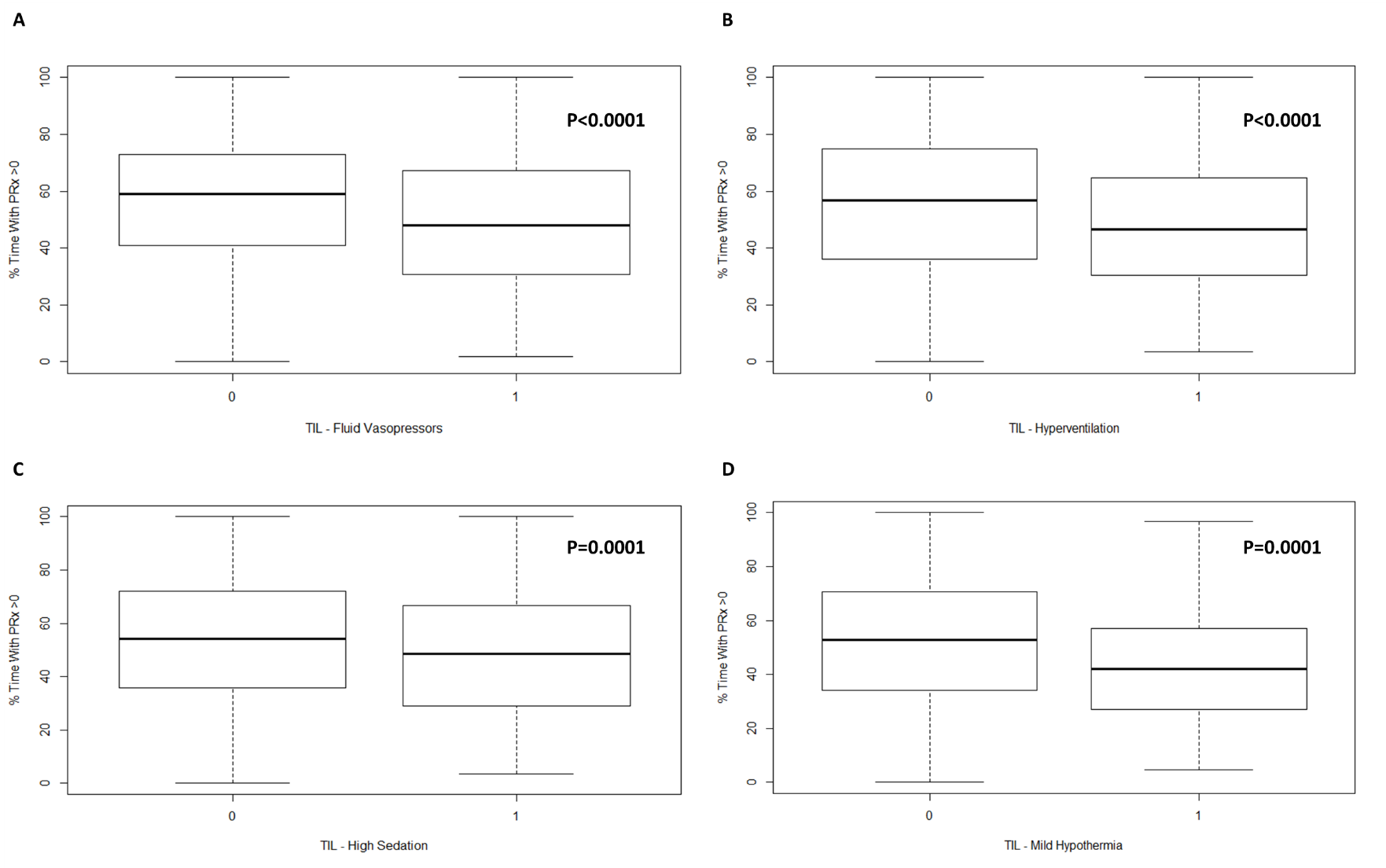
**

*CPP = cerebral perfusion pressure, ICP = intra-cranial pressure, IQR = inter-quartile range, MAP = mean arterial pressure, PRx = pressure reactivity index (correlation between slow-waves in ICP and MAP), SD = standard deviation, TIL = therapeutic intensity level. Figure reports p-values from Mann-U testing, comparing mean daily % time above PRx of 0 for specific TIL Sub-Scores. Figures reports the time-shifted data sheet, evaluating the difference in median values (and IQR) between those receiving a specific intervention vs. those who did not. *NOTE: This p-value is not significant after Bonferroni correction. TIL Fluid Vasopressor – refers to the need for vasopressor therapy to maintain CPP goals. TIL Hyperventilation Mild – refers to mild hypocapnia for ICP control (PaCO2 = 35 to 40 mmHg). TIL Hypothermia Mild – refers to cooling to no lower than 35 Celsius. TIL Sedation High – refers to high sedation levels aimed at ICP control, but not burst suppression.*

Appendix B: Box Plots of Mean Daily % Time with PRx > 0 for Statistically Significant TIL Sub-Scores – Time-Shifted Data Sheet

**
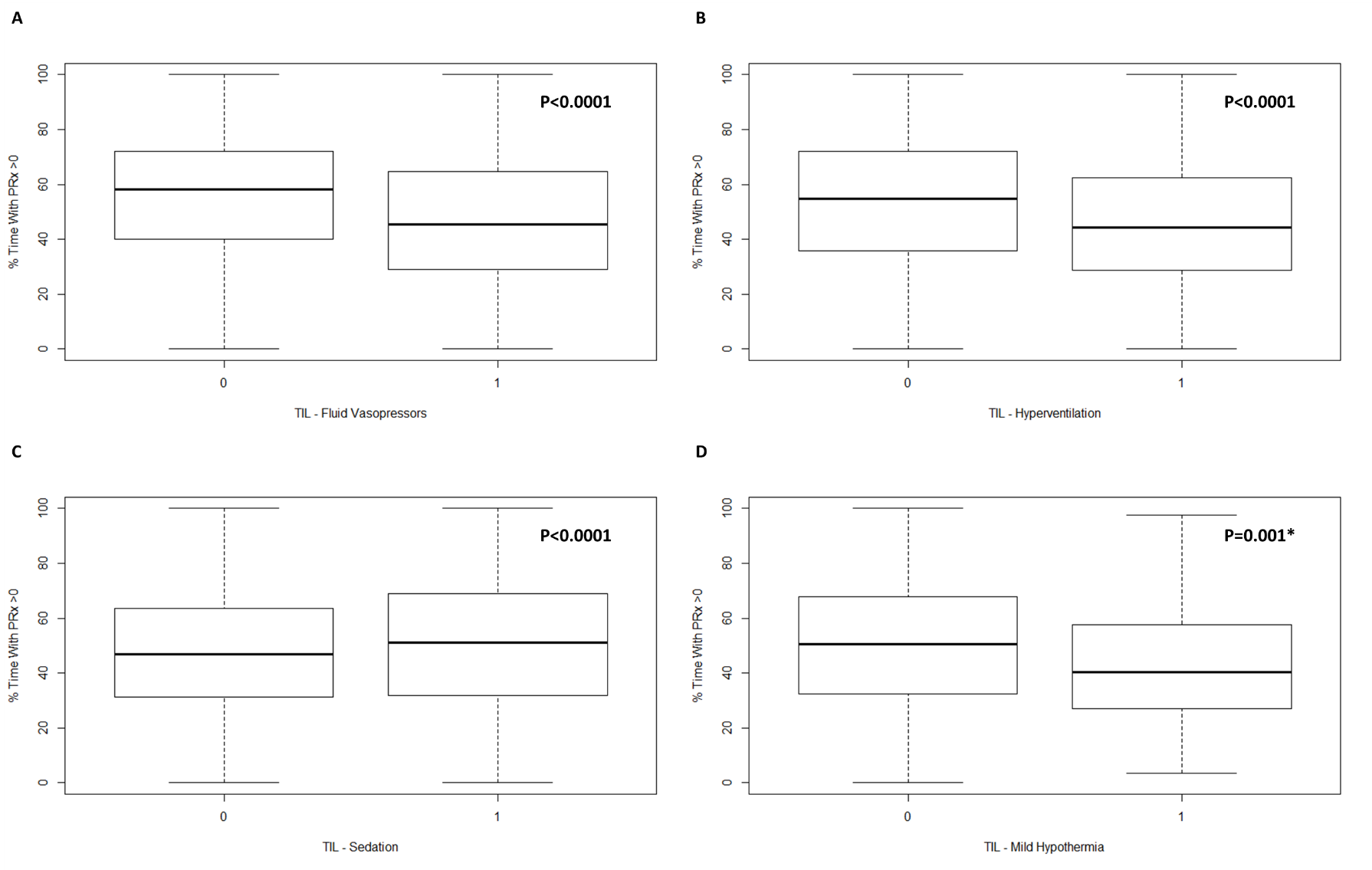
**

CPP = cerebral perfusion pressure, ICP = intra-cranial pressure, IQR = inter-quartile range, MAP = mean arterial pressure, PRx = pressure reactivity index (correlation between slow-waves in ICP and MAP), SD = standard deviation, TIL = therapeutic intensity level. Figure reports p-values from Mann-U testing, comparing mean daily % time above PRx of 0 for specific TIL Sub-Scores. Figures reports the time-shifted data sheet, evaluating the difference in median values (and IQR) between those receiving a specific intervention vs. those who did not. *NOTE: This p-value is not significant after Bonferroni correction. TIL Fluid Vasopressor – refers to the need for vasopressor therapy to maintain CPP goals. TIL Hyperventilation Mild – refers to mild hypocapnia for ICP control (PaCO2 = 35 to 40 mmHg). TIL Hypothermia Mild – refers to cooling to no lower than 35 Celsius. TIL Sedation High – refers to high sedation levels aimed at ICP control, but not burst suppression.
