# Supplementary material for: Cerebrovascular reactivity is not associated with therapeutic intensity in adult traumatic brain injury: a CENTER-TBI analysis
Source: Acta Neurochir (Wien). 2019 Jun 25;161(9):1955–64. doi: 10.1007/s00701-019-03980-8 (PMC6704258; doi:10.1007/s00701-019-03980-8)

**Appendix C: Frequency of Total TIL and TIL Sub-Score Values**

The following histograms represent the population total TIL and TIL Sub-Score values, with reported frequency on the y-axis in number of days for each specific TIL score/sub-score value.

1. Total Daily TIL Score


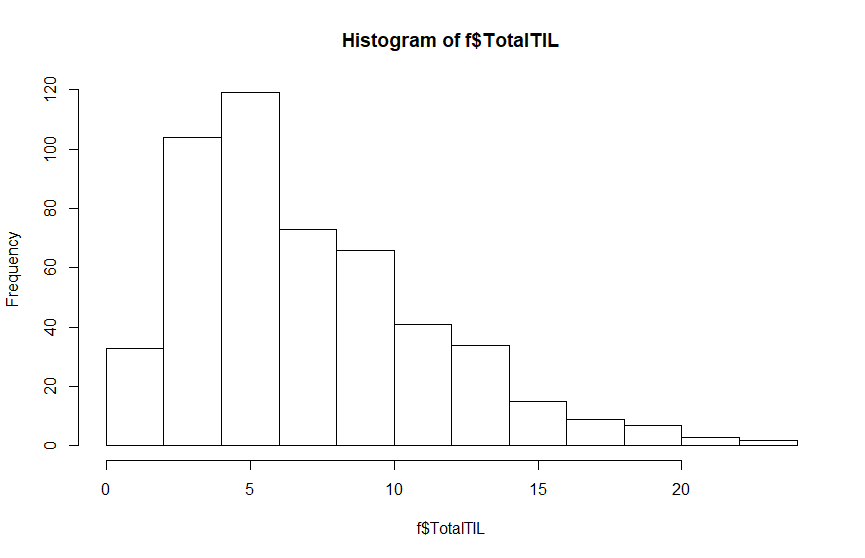


1. TIL Low Hypertonic Saline Dose (no/yes)


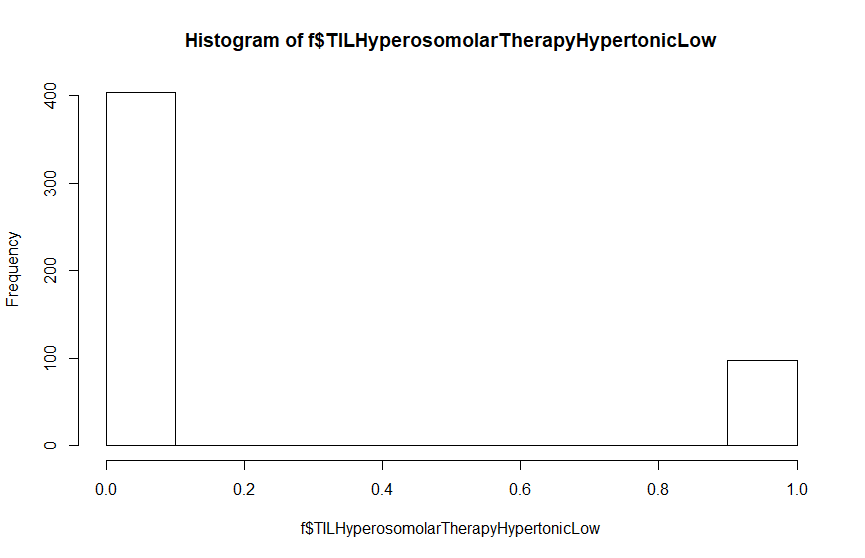


1. High Hypertonic Saline Dose (no/yes)


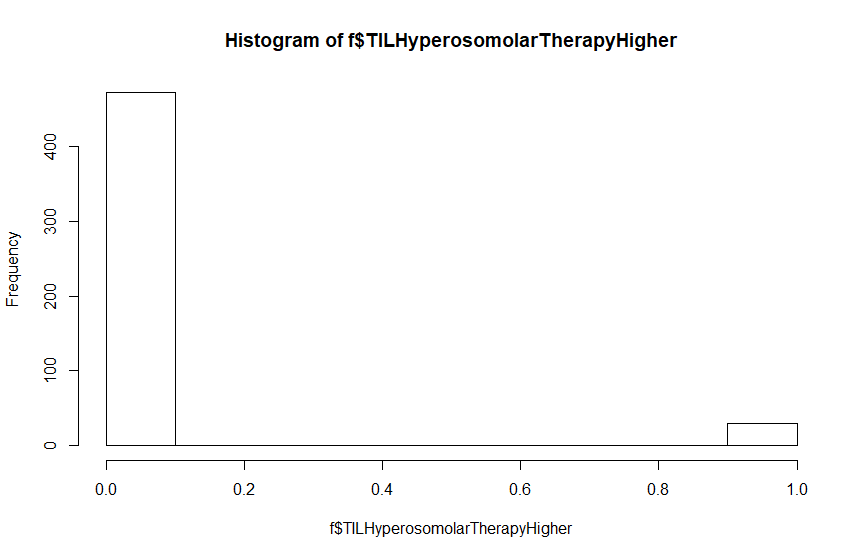


1. Low Mannitol Dose (no/yes)


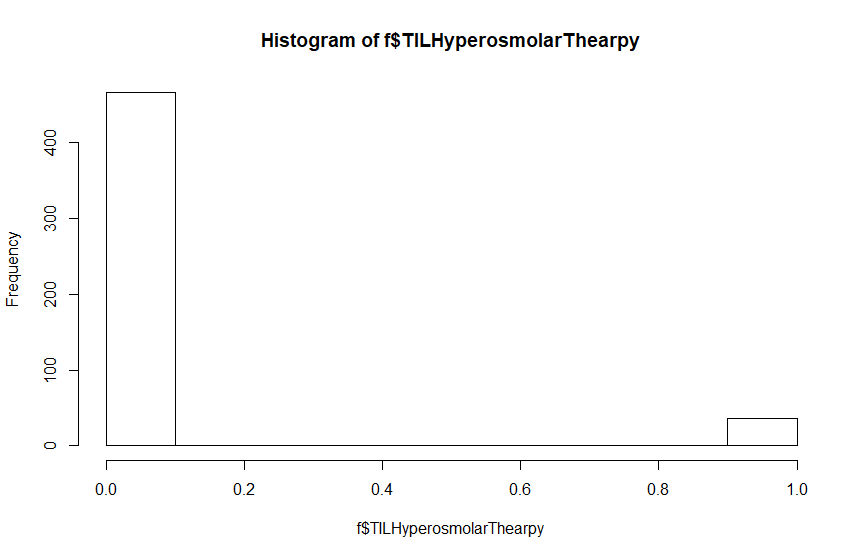


1. High Mannitol Dose (no/yes)


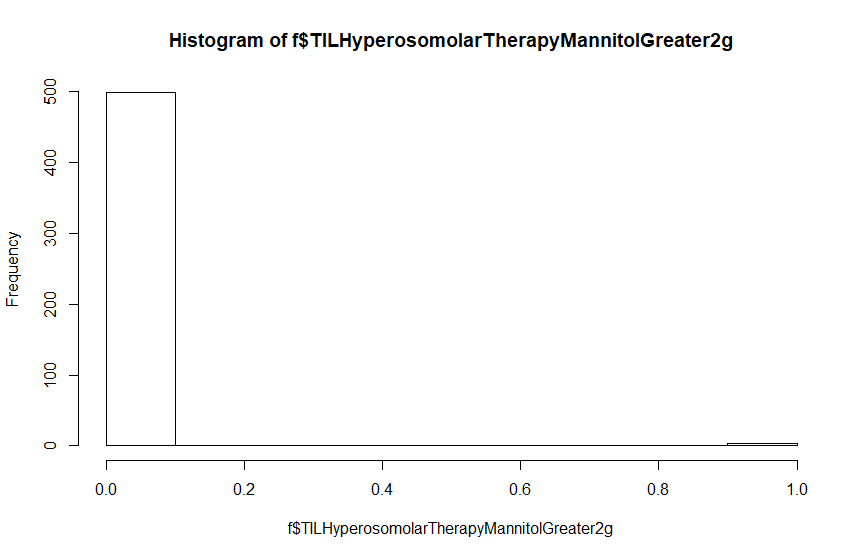


1. Sedation for Ventilation Only (no/yes)


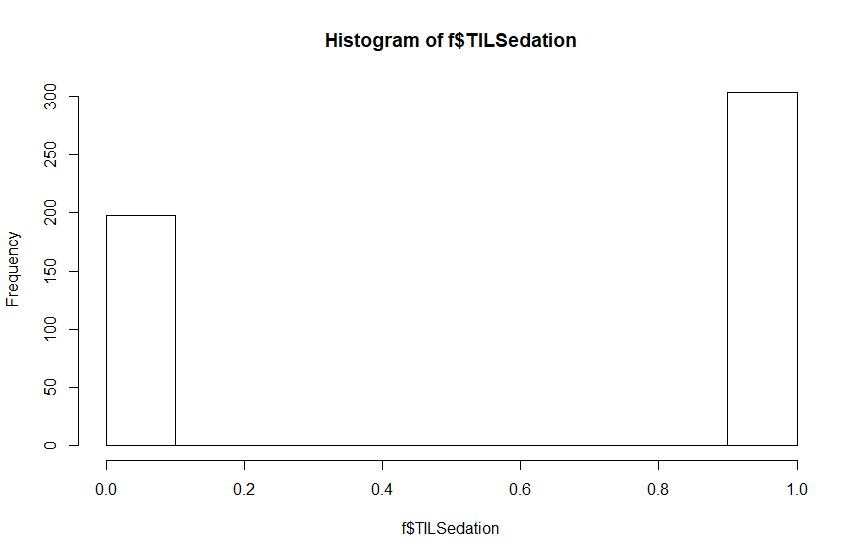


1. Sedation for ICP Control (no/yes)


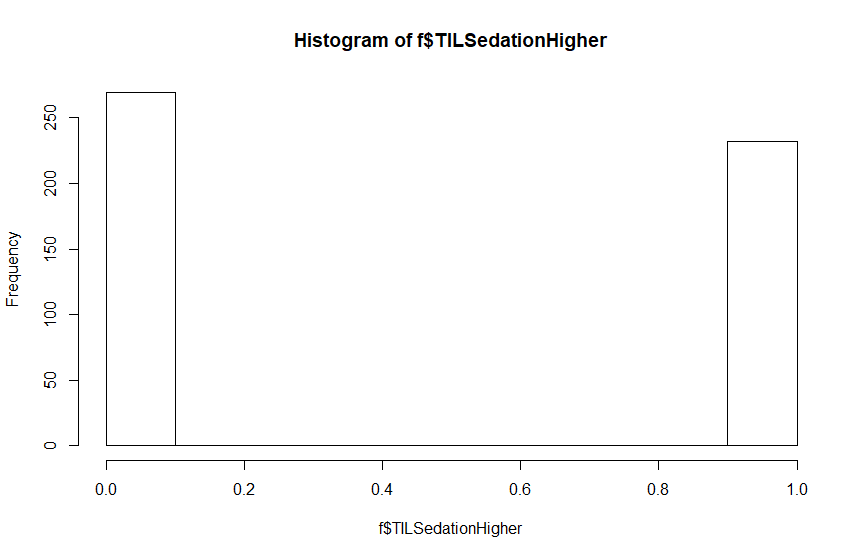


1. Sedation for Metabolic Suppression (no/yes)


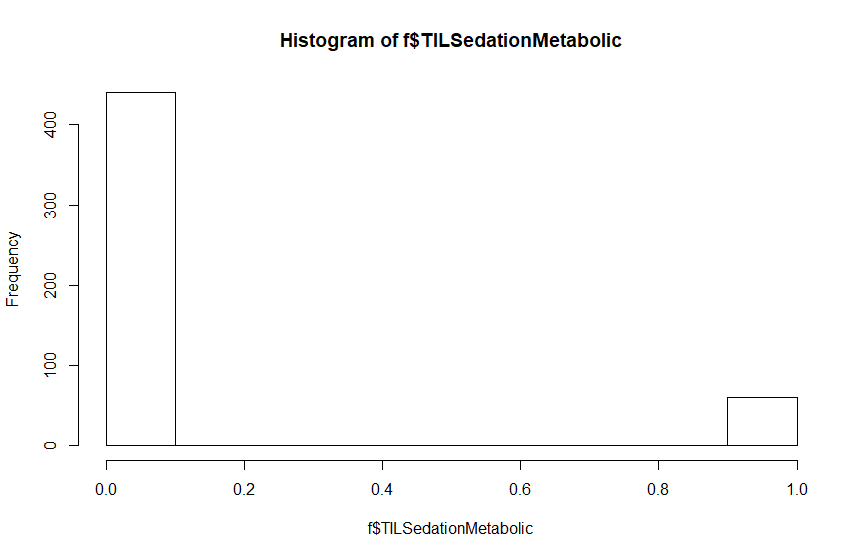


1. CSF Drainage (no/yes)


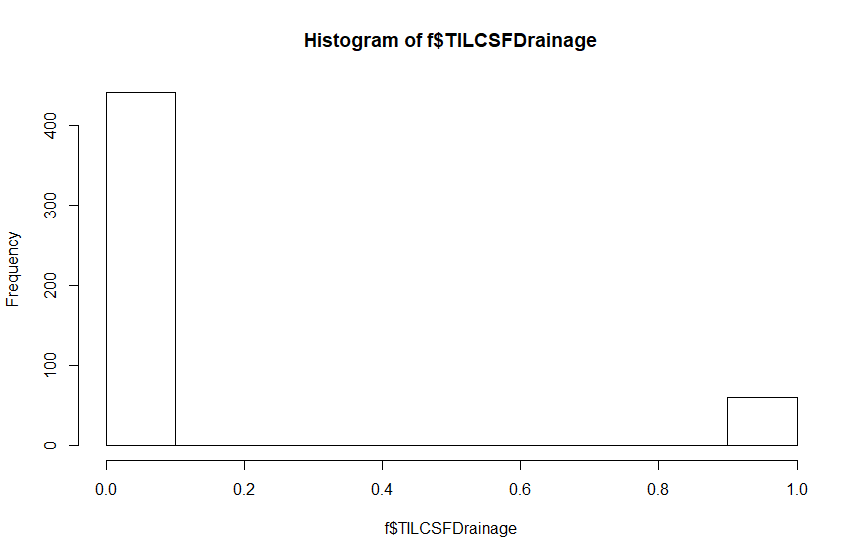


1. Fluid Load for CPP Goals (no/yes)


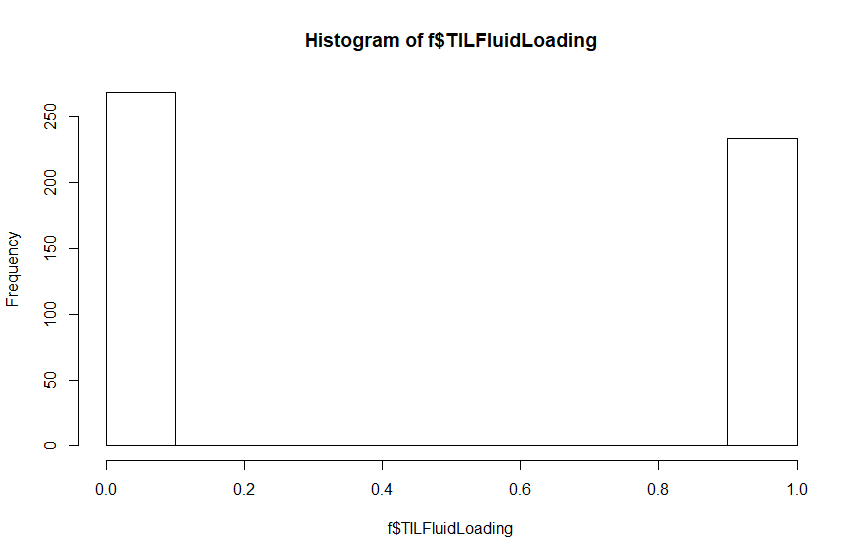


1. Vasopressors for CPP Goals (no/yes)


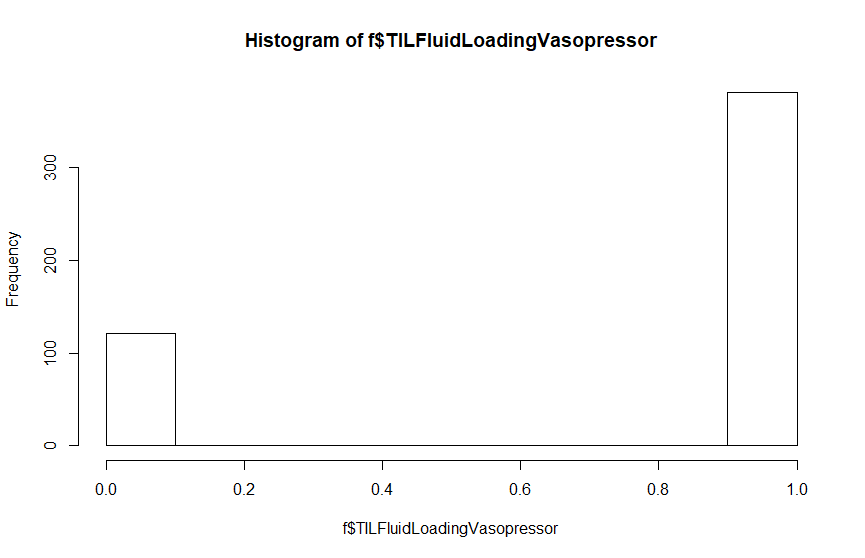


1. Treatment of Fever (no/yes)


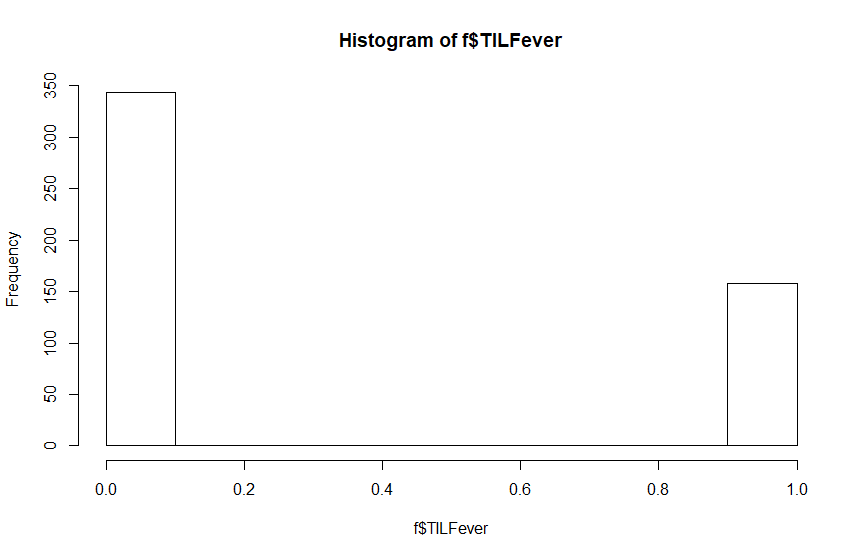


1. Hypothermia Therapy – Mild (no/yes)


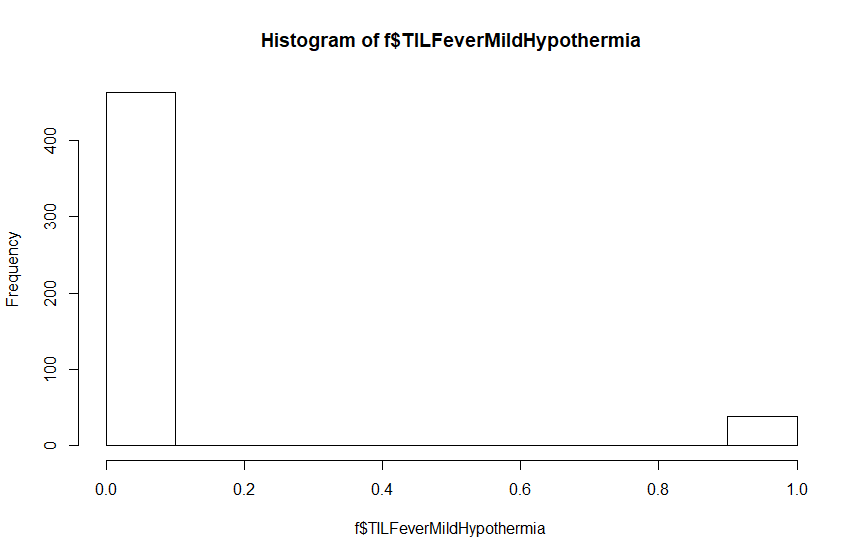


1. Hypothermia – Moderate (no/yes)


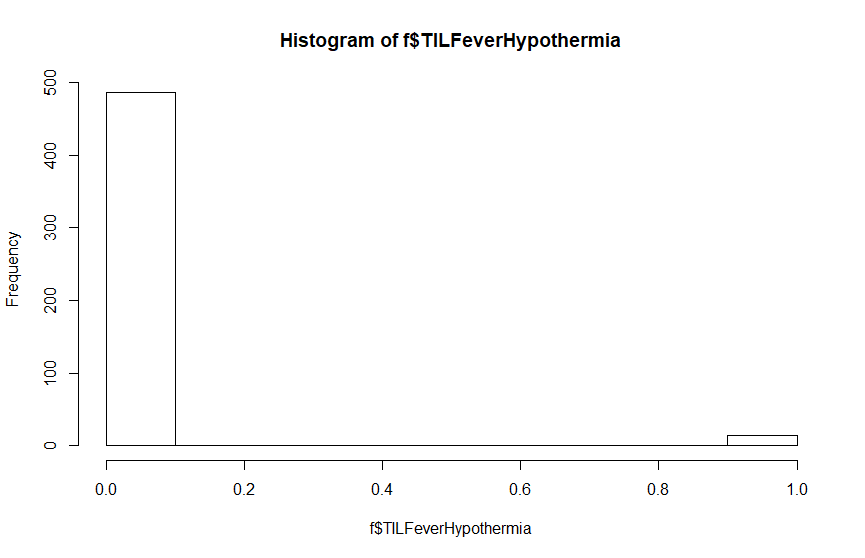


1. Hyperventilation – Mild (no/yes)


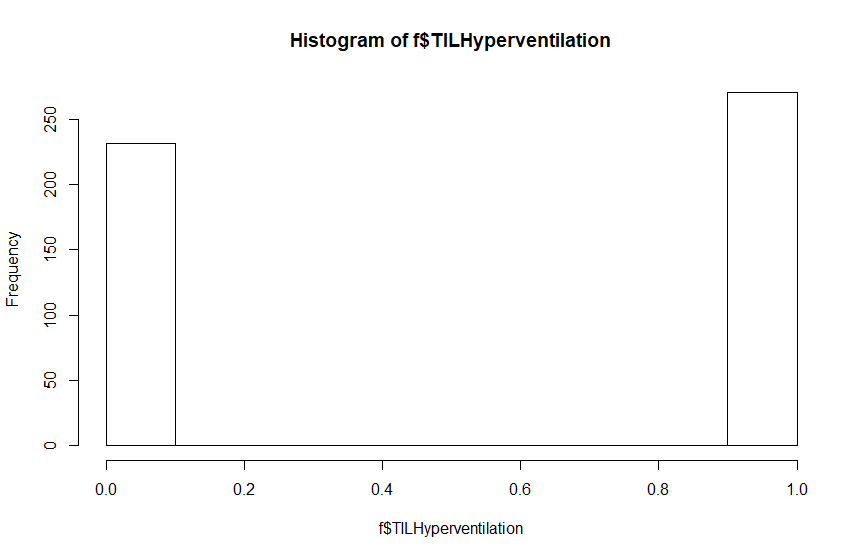


1. Hyperventilation – Moderate (no/yes)


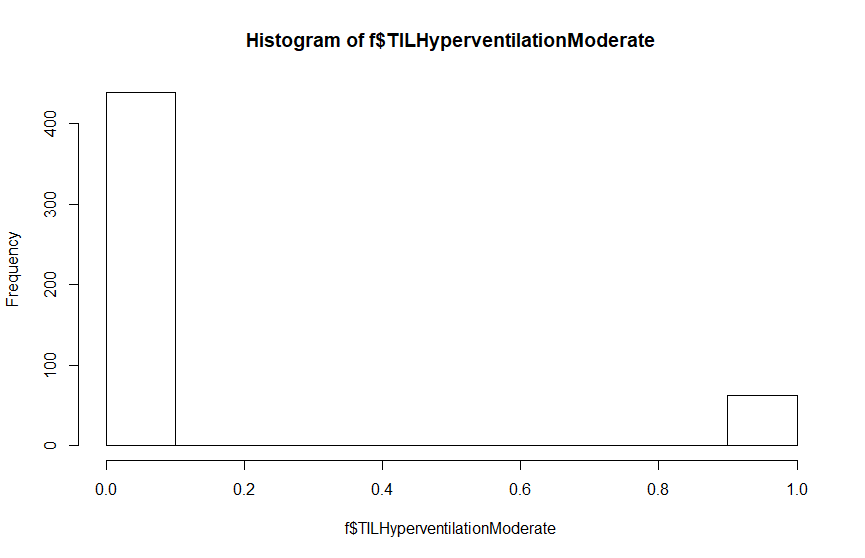


1. Hyperventilation – Intense (no/yes)


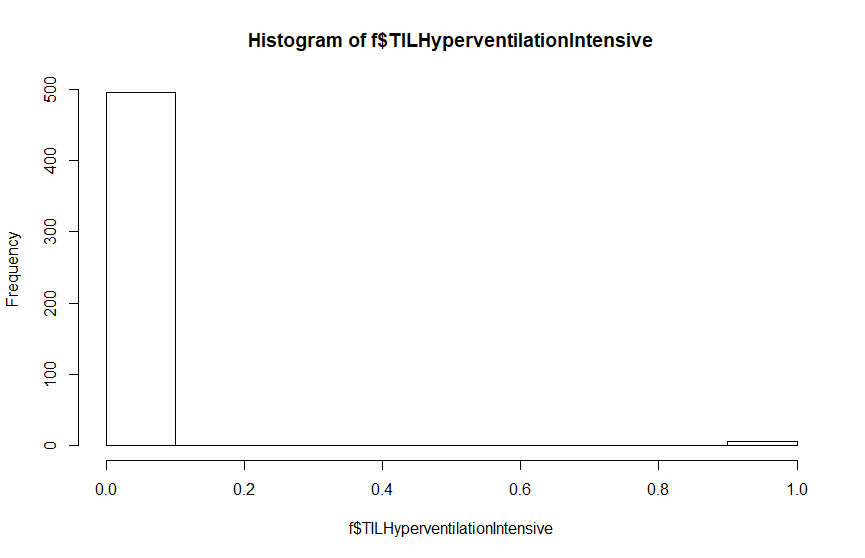


1. Patient Position – HOB Elevated (no/yes)


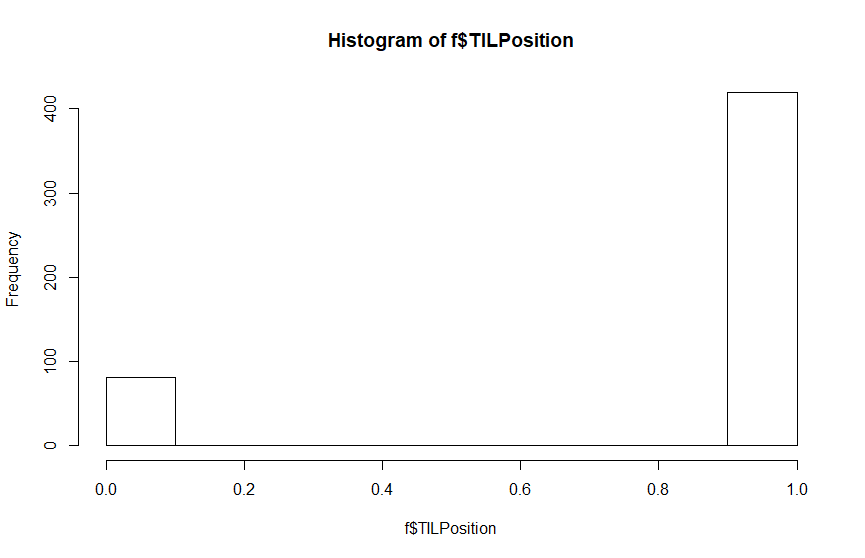


1. Patient Position – Flat (no/yes)


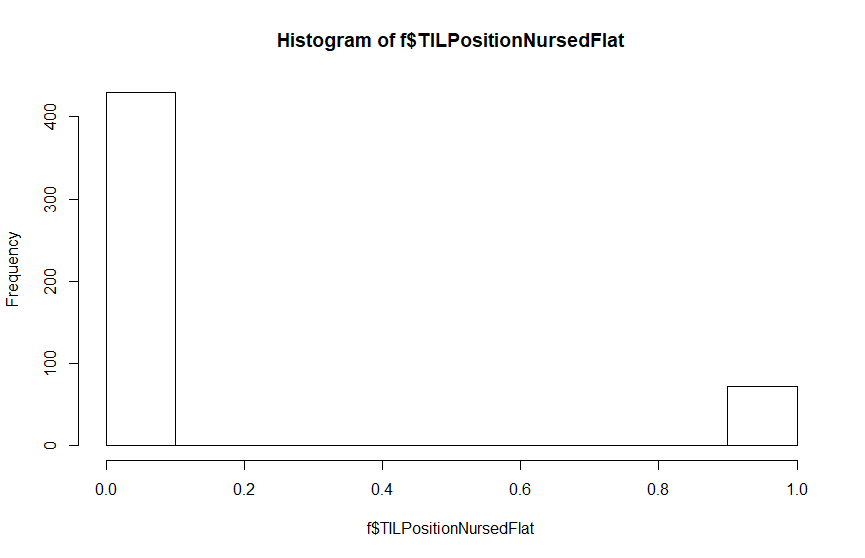


1. Neuromuscular Blockage Used (no/yes)


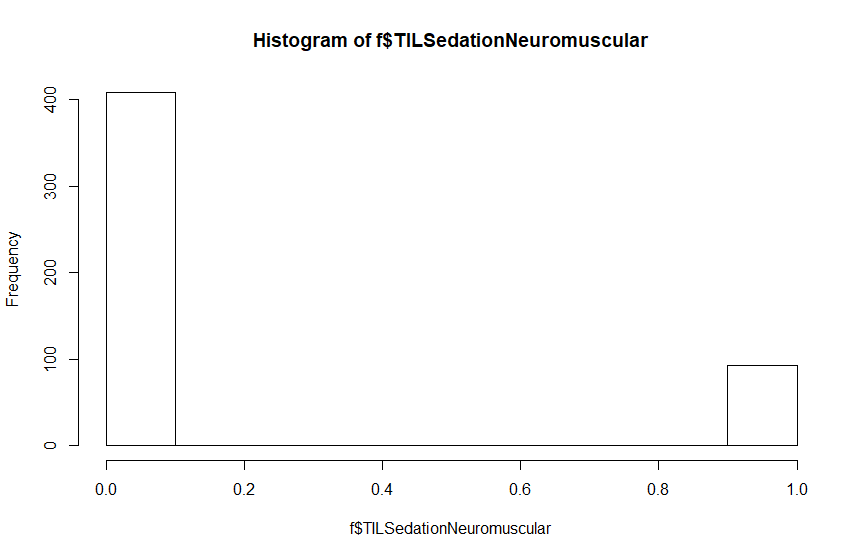


1. Surgery for ICP Control (no/yes)


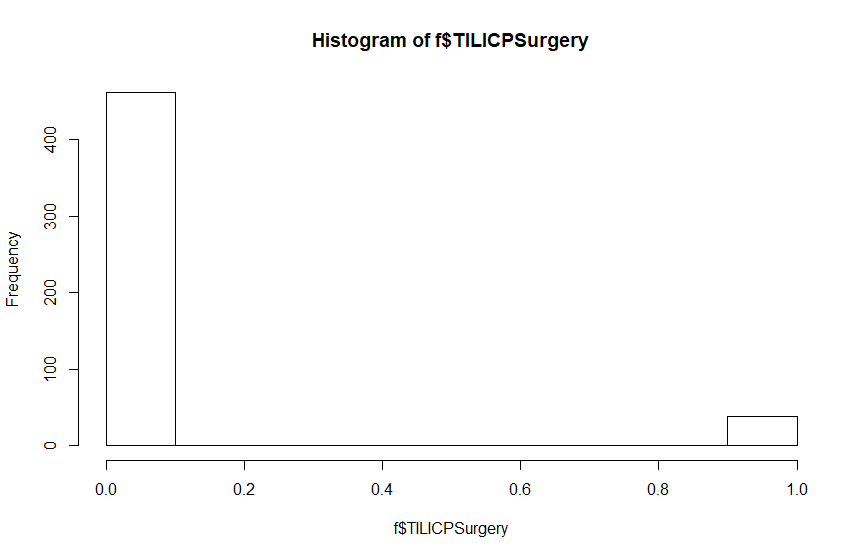


1. Surgery – Decompressive Craniectomy (no/yes)


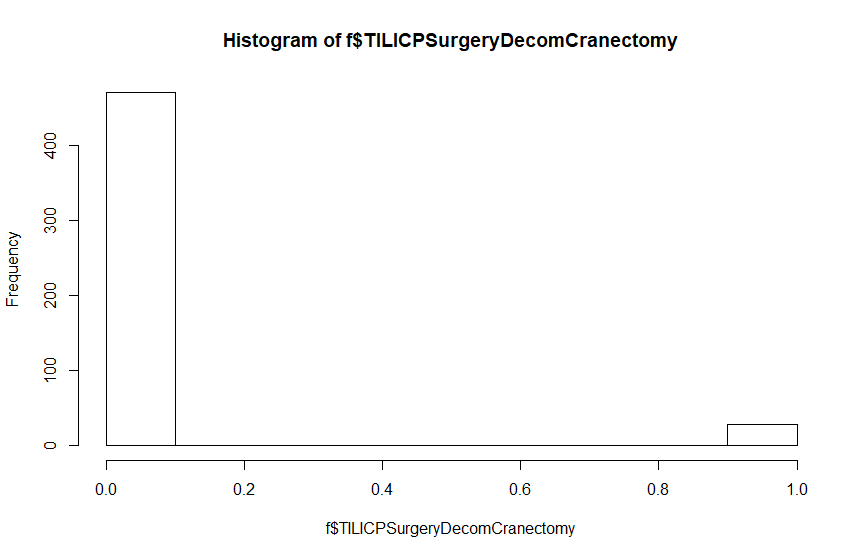

Supplement: Supplementary file 3 — (DOCX 31130 kb). [file 701_2019_3980_MOESM3_ESM.docx]
